# Systemic antibody responses against bacterial and viral antigens associate with fatigue in patients with inflammatory bowel disease

**DOI:** 10.1016/j.isci.2026.116828

**Published:** 2026-07-20

**Authors:** Maximilian G. Griesbaum, Thomas Vogl, Marijn C. Visschedijk, Eleonora A.M. Festen, Gerard Dijkstra, Adina Weinberger, Eran Segal, Rinse K. Weersma, Arno R. Bourgonje

**Affiliations:** 1 Department of Gastroenterology and Hepatology, University of Groningen, University Medical Center Groningen, Groningen, the Netherlands; 2 Center for Cancer Research, Medical University of Vienna, Vienna, Austria; 3 Department of Computer Science and Applied Mathematics, Weizmann Institute of Science, Rehovot, Israel; 4 Department of Molecular Cell Biology, Weizmann Institute of Science, Rehovot, Israel; 5 The Dr. Henry D. Janowitz Division of Gastroenterology, Department of Medicine, Icahn School of Medicine at Mount Sinai, New York, NY, USA

**Keywords:** inflammatory bowel disease, fatigue, antibody responses, humoral immunity, Epstein-Barr virus, influenza, Staphylococcus, PhIP-Seq, biomarkers

## Abstract

Fatigue is common and debilitating in inflammatory bowel disease (IBD), but its etiology remains unclear. Phage-display immunoprecipitation sequencing (PhIP-Seq) was used to profile systemic antibodies against 344,000 rationally selected peptide antigens in 327 patients with IBD, assessing associations with fatigue severity across quartiles and in a remission sub-cohort. No individual antibody remained significant after multiple testing correction. However, aggregation-based analyses identified recurring differences involving Epstein-Barr virus (EBV), influenza virus, and *Staphylococcus* antigens. Higher fatigue was associated with lower responses to EBV early antigen protein D (EA-D) and higher responses to EBV nuclear antigens (EBNA-1, EBNA-3), as well as influenza M1/M2 proteins and *Staphylococcus* clumping factor B. These patterns were not statistically robust and may reflect random variation or disease-/exposure-related factors. Given broader evidence linking EBV-related immune responses to immune-mediated diseases, these observations warrant further investigation. Overall, this study illustrates challenges of linking high-dimensional immune data to complex clinical symptoms.

## Highlights

• Antibodies directed to EBV, influenza, and *Staphylococcus* associate with IBD fatigue
• PhIP-Seq maps antibody repertoires in a high-throughput, high-resolution fashion
• Refined fatigue phenotyping is needed to test disease- and symptom specificity

## Graphical abstract

**Figure:**
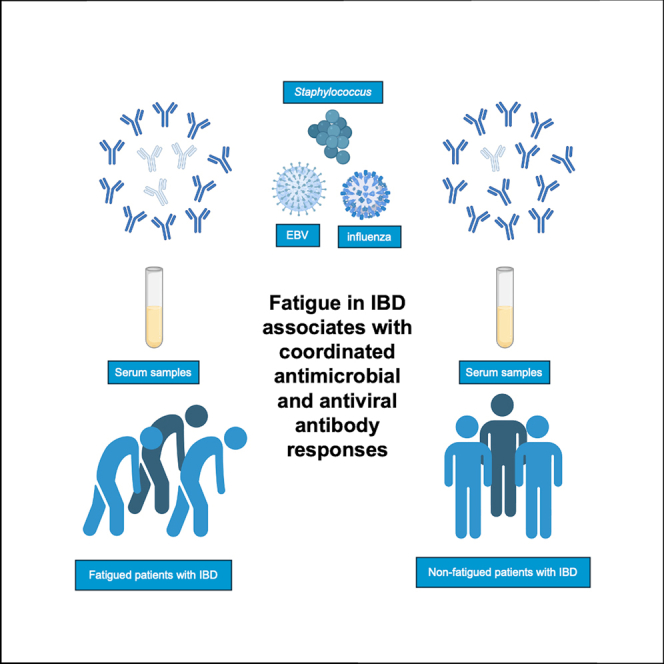

## Teaser

Immunology; Medical microbiology; Computational bioinformatics

## Introduction

Inflammatory bowel diseases (IBDs), encompassing Crohn’s disease (CD) and ulcerative colitis (UC), are chronic and debilitating diseases affecting ∼5 million people globally, characterized by ulcerative inflammation of the gastrointestinal tract.^1^ Although the pathogenesis of IBD is incompletely understood, it entails a complex interplay between genetic susceptibility, the gut microbiome, host immune responses, and environmental (e.g., diet and lifestyle) factors.^2^

The main symptoms of IBD include abdominal pain, diarrhea, rectal bleeding, and fatigue, the latter being a common and clinically challenging symptom affecting up to ∼70–80% of patients with active disease and ∼40–50% of patients with quiescent disease.^3^^,^^4^^,^^5^ Fatigue negatively affects virtually every aspect of life, including energy levels and mental health, and is often accompanied by reduced socioeconomic participation.^5^^,^^6^^,^^7^ Although fatigue is clinically burdensome, its pathophysiology remains heavily understudied. The underlying mechanisms of fatigue in IBD are likely multifactorial, with potential contributing factors including nutritional deficiencies, sleep disturbances, anemia, psychological factors, and subclinical inflammation.^8^^,^^9^

IBD is associated with gut dysbiosis and a decreased diversity of the gut microbiome.^10^ Because of intestinal inflammation, the integrity of the epithelial barrier becomes disrupted, resulting in increased microbial translocation, as well as the entry of small molecules into the underlying tissues and systemic compartment. Furthermore, there seems to be bidirectional communication between the gut and the central nervous system (CNS), which could also be contributing to the development of fatigue in IBD.^11^ Recently, a fecal bacterial signature of fatigue was described in patients with active IBD, who exhibited a less diverse gut microbiome and significant reductions in butyrate-producing bacteria, including *Faecalibacterium prausnitzii* and *Roseburia hominis*, pointing to a potential relevance of the gut microbiome in its development.^12^ Other studies on myalgic encephalomyelitis/chronic fatigue syndrome (ME/CFS) support this, where a decreased stool bacterial diversity has also been identified, suggesting shared pathogenic mechanisms.^13^^,^^14^ In addition to bacteria, viruses also modulate the immune system and are known to trigger the development of a variety of non-communicable diseases, making them possible contributors to fatigue pathogenesis in immune-mediated inflammatory diseases (IMIDs). While triggers related to microorganisms increasingly play a role in the pathogenesis of IMIDs, clear evidence is yet to be found, which warrants further exploration to unravel the potential microorganism-related mechanisms involved in immune responses associated with fatigue in IBD.

Therefore, this study hypothesizes that immune responses against microorganism-related antigens might be involved in the development of fatigue in IBD. Conventional methods such as enzyme-linked immunosorbent assays (ELISAs) can only focus on a single to a few peptides at a time. Recent technological advances have enabled broad, high-resolution investigation of antibody responses. With phage-display immunoprecipitation sequencing (PhIP-Seq), a high-throughput and high-resolution antibody profiling technology, antibody responses against hundreds of thousands of antigen peptides can be investigated in parallel.^15^ Previous studies have successfully used this technology to characterize antibody epitope repertoires in individuals with IBD,^16^^,^^17^ multiple sclerosis,^18^ rheumatoid arthritis,^18^ ME/CFS,^19^^,^^20^ and in viral infections.^21^

Systemic antibody responses in relation to fatigue as a complex clinical phenotype remain insufficiently characterized. Moreover, conventional analyses typically focus on individual antibody associations, which may not capture the high-dimensional structure of immune responses. In this study, we apply high-throughput PhIP-Seq profiling to investigate fatigue in IBD, and we specifically explore coordinated antigen-level antibody patterns as a complementary analytical framework. This approach allows us to generate hypotheses about immune processes underlying fatigue beyond single-antibody associations.

## Results

### Study population characteristics

**Figure 1:**
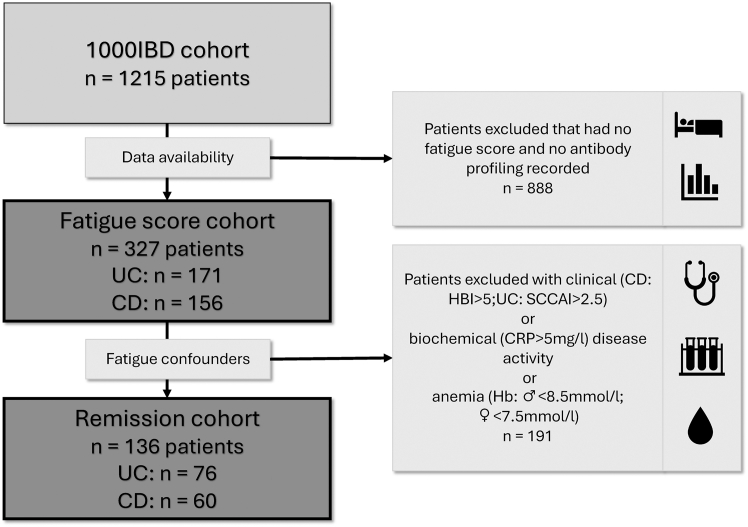
Flow chart of the patient inclusion procedure Patients were initially screened for the availability of fatigue scores^8^ and PhIP-Seq data^16^ at the time of sampling. The resulting patient cohort (*n* = 327) constituted the study population for the present investigation. In addition, we performed stratified analyses based on disease activity and the presence of anemia at time of sampling, factors which are known to be associated with fatigue in IBD.^22^ Here, patients with low clinical disease activity scores (HBI <5 for CD; SCCAI <2.5 for UC), absent systemic inflammation (CRP <5 mg/L), and absent anemia (<8.5 mmol/L for males; <7.5 mmol/L for females) were separated for analysis.

A total of 327 patients were included (Figure 1).^22^ Approximately half of the patients (*n* = 156, 47.7%) had CD and half (*n* = 171, 52.3%) had UC. Notably, there were more women included in the study (*n* = 198, 60.6%). Detailed patient characteristics for each previously defined fatigue group (defined by quartiles, as described in the STAR Methods section) can be found in Tables S1, S2, S3, and S4.

### Patients with CD, smokers, and women reported higher fatigue scores

Median fatigue scores were significantly higher in patients with CD than in patients with UC (6 [4–7] and 5 [4–6], respectively, Mann-Whitney *U*-test, *p <* 0.001). Additionally, smokers were more fatigued compared to non-smokers (6 [5–8] and 5 [4–6], Mann-Whitney *U*-test, *p <* 0.001). Sex differences also became evident, with fatigue scores being slightly higher in women than in men (5 [4–7] and 5 [4–6], Mann-Whitney *U*-test, *p <* 0.001).

### Fatigue associates with clinical disease activity and anemia

**Figure 2:**
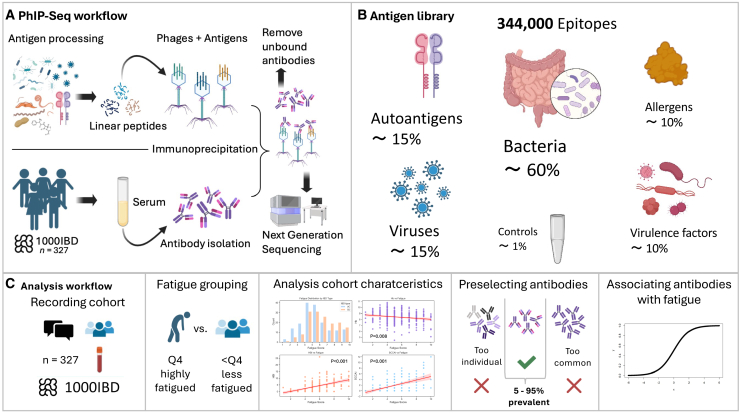
Schematic visualization of the study workflow including data generation and data analysis (A) PhIP-Seq combines circulating antibodies from individuals with synthetic antigen libraries of phages expressing antigens on their surface. Thereby, after immunoprecipitation and next-generation sequencing, the antibody repertoire for each patient can be determined. (B) The antigen libraries used in this study covered a total of 344,000 rationally selected peptide antigens covering bacteria, autoantigens, viruses, allergens, virulence factors, and controls, as previously described in detail.^16^^,^^23^^,^^24^^,^^25^ (C) After recording patient data, patients were grouped based on their self-reported fatigue scores, and patient characteristics were analyzed. Antibody prevalence (>5% and <95% in the entire cohort) was filtered, and data analysis was performed aiming to identify specific antibody responses associated with fatigue. Abbreviations: IP, immunoprecipitation; NGS, next-generation sequencing.

For a visual summary of the study workflow, see Figure 2. Figure 2A shows the PhIP-Seq workflow, Figure 2B shows the antigen library composition and Figure 2C shows the analysis workflow. Before analyzing specific antibody responses associated with fatigue in IBD, fatigue scores were first compared across different levels of clinical disease activity, since this is commonly reported to be linked with fatigue in IBD.^22^ Indeed, a higher clinical disease activity score was associated with higher fatigue, as there was a moderate positive correlation between fatigue scores and clinical disease activity score in both CD and UC (HBI: ⍴ = 0.57; *p <* 0.001; SCCAI: ⍴ = 0.49; *p <* 0.001). In addition, hemoglobin showed a weak inverse association with fatigue scores (⍴ = −0.14; *p* = 0.008).

These findings led to the selection of a sub-cohort without anemia and in clinical and biochemical remission in which all analyses were repeated (Figure 1).

### Patients with IBD in remission still report high fatigue scores

The sub-cohort consisting of patients with quiescent IBD reported slightly, but significantly lower fatigue scores (5 [4–6]) than patients with active disease and/or anemia (5 [4–8]) (Mann-Whitney *U*-test, *p <* 0.001). However, more than half (51%) of these patients still reported being at least moderately fatigued (fatigue score ≥5), which was not far from those with active disease and/or anemia (73%). These findings agree with previous studies recording fatigue in about 50% of patients in remission.^9^

After having established these confounders, PhIP-Seq data were linked to fatigue scores and subsequently different fatigue groups using multivariable logistic regression analysis adjusted for age, sex, and smoking status.

### No individual peptides, large antigen groups, or previously discovered ME/CFS-associated peptides showed significant associations with fatigue

Antibody-bound peptide frequencies were compared between fatigue groups (Q4 vs. < Q4) using logistic regression adjusted for age, sex, and smoking.

Across all analyses, including those stratified by IBD type (UC, CD), remission status, and alternative fatigue group definitions (Q1 vs. Q4, Q1+Q2 vs. Q3+Q4, Q1 vs. > Q1), no single peptide alone remained significant after false discovery rate correction (Tables S5, S6, S7, S8, S9, S10, S11, S12, S13, S14, S15, S16, S17, S18, S19, S20, S21, S22, S23, S24, S25, S26, S27, and S28).

To explore potential higher-level effects, peptides were collapsed into eight prespecified antigen libraries (IEDB, gut microbiome, VFDB, pathogenic bacteria, phages, antibody-coated bacteria, probiotic bacteria, and allergens). Although a few libraries reached nominal significance in the primary contrast (*p <* 0.05), none survived FDR correction (*q* ≥ 0.05), and directions were inconsistent across analyses and disease strata.

Finally, overlap with previously reported ME/CFS-associated peptides^19^ was tested, including restriction to flagellin-directed peptides. However, no peptide remained significant after multiple testing correction (all *q* ≥ 0.05; Tables S29, S30, S31, S32, S33, S34, S35, S36, S37, S38, S39, and S40).

### Fatigue in IBD is potentially associated with systemic antibody responses directed at viral and bacterial antigens

**Figure 3:**
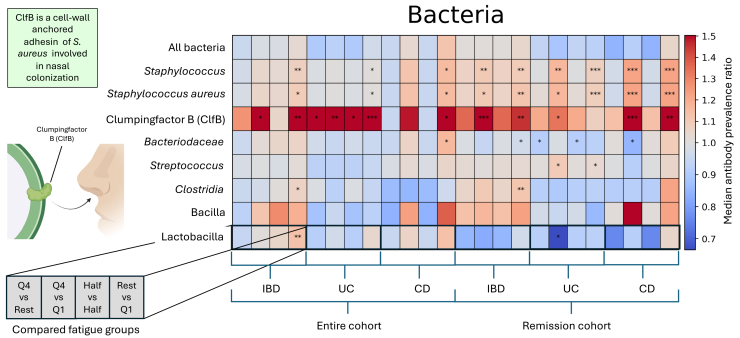
Viral antibody responses associated with fatigue in IBD (A) Heatmap shows increased (red) and decreased (blue) median antibody prevalences of an antibody group. For antigens represented by multiple antibodies (e.g., EBNA-1), prevalence was calculated per antibody within each fatigue group, and the median prevalence across antibodies targeting the same antigen was used for visualization and then compared between fatigue groups (Mann-Whitney *U*-test; Asterisks mark adjusted significance (∗∗∗ = *q* < 0.001; ∗∗ = *q <* 0.01; and ∗ = *q* < 0.05); scatterplots show the strongest prevalence increase in antibodies directed at EBNA-1 in all patients (B), decrease in antibodies directed at EA-D (BMRF-1) in patients with CD (C), increase in antibodies against EBNA-3A in patients with UC (D) in more fatigued groups). Abbreviations: IBD, inflammatory bowel disease; UC, ulcerative colitis; CD, Crohn’s disease; EBV, Epstein-Barr virus; HHV, human herpes virus.

**Figure 4:**
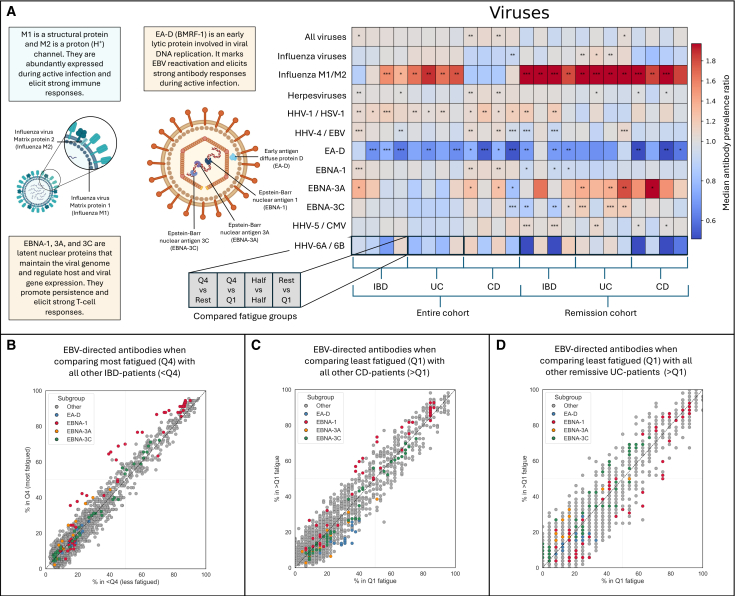
Bacterial antibody responses associated with fatigue in IBD Heatmap shows increased (red) and decreased (blue) median antibody prevalences found in analyses comparing different fatigue groups (Mann-Whitney-*U* test; Asterisks mark adjusted significance (∗∗∗ = *q* < 0.001; ∗∗ = *q <* 0.01; and ∗ = *q <* 0.05)). Abbreviations: IBD, inflammatory bowel disease; UC, ulcerative colitis; CD, Crohn’s disease; ClfB = clumping factor B.

Although logistic regression in these analyses did not yield significant associations after correcting for multiple comparisons, grouping antibodies by antigen taxonomy revealed antigen-specific changes in antibody frequencies. Figures 3 and 4 show heatmaps that visualize the most recurring antibody prevalence alterations across the different comparisons.

### EBV and influenza antibody patterns in fatigued IBD

Across different fatigue group comparisons, generally viral antibody responses among more fatigued groups of patients with IBD (e.g., Q4 compared to the rest) were characterized by a decrease in antibodies directed at the EBV lytic antigen BMRF-1, an increase in responses against EBV latent nuclear antigens EBNA-1 and EBNA-3, and an increase in responses against influenza proteins M1 and M2 (Figure 3).

The most evident antibody prevalence change observed was a significant decrease in antibodies directed at the BMRF-1 antigen of EBV in fatigued groups. More than half of the analyses (62.5%) showed a significant (*q <* 0.05) decrease in antibodies directed at BMRF-1 in the more fatigued groups. This difference was most prominent in patients with active CD, where the median antibody prevalence in the more fatigued group (>Q1) was around half that of the least fatigued group (Q1) (14.7% vs. 31.1%, Mann-Whitney *U*-test, *q <* 0.001). This association was also seen in quiescent CD, but not in UC, where the association also showed a slight decrease in the more fatigued groups, but this difference was non-significant (*q >* 0.05).

Another notable pattern involved antibodies directed against the EBV nuclear antigens EBNA-1, EBNA-3A, and EBNA-3C. For EBNA-1, antibodies were slightly more prevalent in the most fatigued group (Q4) of patients with CD, compared to the less fatigued group (<Q4), with median prevalences of 54.7% and 45.2%, respectively (Mann-Whitney *U*-test, *q <* 0.05).

Conversely, antibodies against EBNA-3A showed an increase, most evident in patients with quiescent UC, where more fatigued individuals (>Q1) had a significantly higher median prevalence (36.8%) compared with the least fatigued group (Q1; 25.9%) (*q <* 0.01). EBNA-3C antibodies displayed opposing patterns across disease groups: In patients with CD, antibody prevalence was lower in more fatigued groups (>Q1) than in least fatigued (Q1) (median prevalence ratio = 0.72, *q <* 0.05), whereas in quiescent UC, the opposite trend was observed, with a higher prevalence in the most fatigued group (Q4) compared with the remainder (<Q4) (median prevalence ratio = 1.42, *q <* 0.01).

Finally, another notable viral antibody pattern was observed for influenza M1/M2 proteins. Antibodies directed against influenza M1/M2 were 1.7–1.9 times more common in fatigued groups with active UC (Mann-Whitney *U*-test, *q <* 0.05), whereas in fatigued groups with active CD, a slight but non-significant decrease (median FC: 0.8–1.1) was observed. This significant increase was even stronger during remission, where antibodies were more than twice as common in fatigued groups with UC (median FC: 2.1–2.7) and in fatigued groups with CD (median FC: 1.8–2.5) than in the less fatigued groups.

### Potentially increased antibody responses against clumping factor B (ClfB) of *Staphylococcus aureus* in fatigued patients with IBD

Bacterial antibody responses in fatigued patients with IBD were characterized by an increase in antibody responses against *Staphylococcus aureus* clumping factor B (ClfB). This was most evident in groups with active UC (median FC: 1.5–1.6) (Mann-Whitney *U*-test, *q <* 0.001) but was not confirmed in groups with quiescent UC. In fatigued groups with CD, on the other hand, while responses during active disease were unchanged, an increase was seen in groups with quiescent CD. However, this was less consistent across group comparisons ranging from non-significant differences for example, when comparing the most fatigued (Q4) remissive patient group with CD versus the group with the rest of the remissive patients with CD, to up to 3.2 times more common in the group with the most (Q4) fatigued remissive patients with CD when comparing to the group with the least fatigued (Q1) remissive patients with CD instead (Mann-Whitney *U*-test, *q <* 0.001).

### Permutation-analysis

Permutation-based analyses were performed to assess whether the observed aggregation-level patterns exceeded random structure. Across all patients, the global aggregation statistic was not significantly different from the null distribution (*p* = 0.44), indicating that comparable signal magnitudes frequently arose under random label assignment.

Targeted analyses of selected antigen groups, including EBV EA-D, EBNA-1, influenza M1/M2, and *Staphylococcus* ClfB, similarly did not demonstrate evidence of non-random structure (all *p* > 0.1).

## Discussion

This exploratory study identified recurring antigen-level antibody response patterns across fatigue groups in patients with IBD. In an unbiased large-scale analysis, antibody epitope repertoires of 327 individuals were modeled across different levels of fatigue. Importantly, this study differs from previous work by focusing on fatigue as a clinical phenotype rather than disease status alone, and by leveraging high-dimensional antibody profiling to explore coordinated immune response patterns rather than single-antigen associations. Although no individual peptide remained significant after multiple testing correction, aggregation-based analyses repeatedly highlighted EBV-, influenza-, and *Staphylococcus*-related antigens among the strongest signals. Given the absence of peptide-level significance and the non-significant permutation analyses, these findings should be interpreted cautiously and primarily as hypothesis-generating observations.

### Antigen-specific antibody responses directed at EBV potentially associate with fatigue in IBD

Among the recurring aggregation-level signals, EBV-related antigens were among the most prominent. A lower prevalence of antibodies targeting BMRF-1 was observed in groups with more fatigued patients, particularly in patients with active CD. However, this decrease was less pronounced in quiescent CD and largely absent in UC. In contrast, antibodies against EBV nuclear antigens showed different patterns across CD and UC subgroups. EBNA-1 antibodies were more prevalent in groups with fatigued patients with CD, while EBNA-3A and EBNA-3C responses were higher in groups with fatigued patients with UC, especially in quiescent UC. These patterns are of potential interest, but they do not establish fatigue-specific EBV involvement and should be interpreted as exploratory associations within an IBD cohort.

EBV is a common herpesvirus with a lifetime prevalence of 90–95% in adults worldwide, establishing lifelong latency in human B cells.^26^ Among the EBV proteins analyzed, BMRF1, EBNA-1, and EBNA-3 represent distinct stages of viral activity in lytic and latent phases. EBNA-1 is a latent EBV antigen with well-described roles in viral persistence and host immune recognition.^27^^,^^28^^,^^29^ The increased prevalence of EBNA-1-directed antibodies in groups with patients with active CD may be consistent with the altered immune recognition of latent EBV antigens, although the current data do not allow inference about the underlying mechanism. EBNA-3C is likewise linked to latent EBV biology and immune recognition.^30^ The higher EBNA-3C antibody prevalence observed in groups with fatigued patients with quiescent UC may point to differences in EBV-related immune memory or antigen exposure,^31^ but the present data cannot distinguish between latent, lytic, or other virological states. Likewise, the decrease in antibodies directed against the early lytic antigen BMRF1 may indicate differences in the immune recognition of EBV lytic antigens but should not be taken as evidence for a specific stage of viral activity.^26^^,^^30^ Altogether, these findings may indicate that fatigue in IBD is accompanied by differences in EBV-related antibody patterns. However, because viral activity, T cell responses, and cytokine signaling were not directly measured, any mechanistic interpretation remains speculative.

Comparable alterations in EBV-specific immunity have been reported across other chronic immune-mediated diseases. Notably, EBV has also been implicated as a potential trigger of fatigue-related conditions such as ME/CFS, with prospective studies showing that acute EBV infectious mononucleosis can precipitate long-lasting fatigue syndromes in a subset of individuals.^32^^,^^33^^,^^34^ Likewise, longitudinal serological studies have established EBV as an important factor in the development of multiple sclerosis, underscoring its broader role in chronic immune dysregulation.^35^ Recent work has further highlighted the role of Epstein-Barr virus (EBV) in immune-mediated diseases, particularly in the context of latency-lytic dynamics, their potential contribution to chronic immune dysregulation, and how variation in EBV persistence and immune recognition may influence autoimmune disease risk.^36^^,^^37^ Two large-scale sero-epidemiological studies extended these links to IBD. In a U.S. military cohort using the VirScan platform, individuals with prior EBV exposure were reported to be approximately three times more likely to develop CD, and EBNA-1 seropositivity and antibody titers were significantly elevated up to seven years before diagnosis, suggesting early viral immune activation.^38^ In parallel, a European PhIP-Seq study revealed distinct pre-diagnostic antibody signatures in both CD and UC, including divergent patterns in EBV-directed responses.^17^ Interestingly, they reported reduced antibody responses directed at EBNA-1 and the capsid protein VP26, alongside increased responses against EBNA-3C in patients after IBD onset. In contrast, our data reveal an increased prevalence of antibodies directed at EBNA-1 and EBNA-3A/3C depending on IBD type, accompanied by a consistent decrease in BMRF1 reactivity in groups with fatigued patients. Together, these observations suggest that EBV-related antibody patterns linked to fatigue within established IBD may differ from EBV-related patterns observed around disease onset. However, the present data do not allow conclusions about the virological or immunological mechanisms underlying these differences.

Another recent study investigating antibody responses in individuals with established disease also reported distinct EBV-specific antibody shifts in CD and UC compared with healthy controls (HC).^16^ Here, CD was characterized by increased reactivity to the lytic trans activator BZLF1 compared to controls, while EBV nuclear antigen signals were more prominent in UC, with EBNA-1 peptides showing higher reactivity and an EBNA-3B/EBNA-4-derived peptide showing lower reactivity compared with HC. Comparisons between CD and UC additionally showed higher EBNA-1 reactivity in CD. The findings of the current study show a related pattern in the context of fatigue. EBNA-1 antibodies were increased in groups with fatigued individuals with CD, whereas EBNA-3A and EBNA-3C responses were more prevalent in groups with fatigued individuals with UC; in addition, both diseases showed lower BMRF1 reactivity in groups with more fatigued patients. This suggests that some EBV-related antibody patterns observed in established CD and UC may also be present across fatigue groups within these diseases, whereas the lower BMRF1 reactivity observed here warrants further study but cannot be considered fatigue-specific based on the current study design.

### Additional antigen-specific antibody responses directed at matrix proteins of the influenza virus potentially associate with fatigue in IBD

Beyond EBV, a consistent antibody pattern was also observed for influenza virus matrix proteins M1 and M2. In patients with active UC, antibodies directed at influenza M1/M2 were roughly twice as prevalent in fatigued groups with active disease, and this increase was even more pronounced in quiescent disease. In CD, antibody prevalences showed a less consistent pattern, with a slight but non-significant decrease in active disease and a strong elevation in quiescent disease. The M1 protein forms the structural scaffold of the virion and controls viral ribonucleoprotein (vRNP) export and assembly, whereas M2 acts as a proton-selective ion channel mediating viral uncoating and pH-dependent maturation.^39^ Its conserved extracellular M2e domain elicits cross-reactive antibodies that are leveraged in vaccine design and mediate Fc-effector-based protection.^40^^,^^41^^,^^42^

No human studies have directly linked antibody responses to M1 or M2 with fatigue in IBD or after influenza infection. Clinical research on M2e has focused on prophylaxis and disease severity rather than fatigue phenotypes.^43^^,^^44^ In IBD, immunosuppressive therapy can attenuate antibody responses to influenza vaccination, suggesting preserved yet modulated antiviral immunity.^45^ Fatigue commonly follows viral infections, and cohort studies indicate that influenza infection may increase long-term fatigue risk.^34^^,^^46^ However, large registry and clinical trial data show no association between influenza vaccination and chronic fatigue syndrome.^47^^,^^48^

Taken together, the increased antibody responses against M1/M2 proteins in fatigued patients with IBD may suggest dysregulated antiviral memory responses, particularly in UC, consistent with broader immune activation contributing to fatigue. Future studies integrating detailed fatigue phenotyping with multiplex viral antibody profiling factoring in previous exposure history and confounding medications are needed to clarify whether influenza-specific immune memory contributes to systemic symptoms in IBD.

### Systemic antibody responses directed at bacterial antigens are characterized by responses directed at *Staphylococcus* clumping factor B (ClfB)

In addition to viral antibody responses, groups with more fatigued patients also showed enrichment of antibodies against the *Staphylococcus aureus* virulence factor clumping factor B (ClfB), especially in active UC. ClfB is an adhesin that binds epithelial ligands such as loricrin and cytokeratin 10, facilitating nasal and skin colonization.^49^ At these sites, *Staphylococcus* antigens can promote inflammation. Although *Staphylococcus* bacteria are not classically involved in IBD pathogenesis, compromised epithelial integrity in active IBD could permit the translocation of bacterial antigens through the intestinal epithelium into the systemic circulation.^50^^,^^51^^,^^52^ Mechanistically, *Staphylococcus aureus* superantigens can activate large numbers of T cells and induce a systemic cytokine surge, amplifying mucosal inflammation and potentially contributing to cytokine-driven fatigue.^53^^,^^54^ Therefore, an increased anti-ClfB response in fatigued patients with IBD could be a signature of staphylococcal infection elsewhere that may promote fatigue through systemic inflammation. However, these findings did not survive permutation analysis and should be interpreted with caution.

### No fatigue-related antibody response directed at bacterial flagellins

This study explored antibody associations of fatigue in patients with IBD, a population in which previous studies have established the presence of increased antibody-directed responses compared to healthy individuals against bacterial flagellins, especially those belonging to the family of *Lachnospiraceae*.^16^^,^^55^ However, in the current study, no significant differences in antibody responses against bacterial flagellins were observed across different levels of fatigue within patients having IBD. Previous work has shown that antibody binding to flagellins is enriched and predominantly targets the N-terminal domain, corresponding to so-called “silent” and “stimulator” flagellins, in both CD and ME/CFS. In CD, additional antibody binding to the C-terminal domain (notably of stimulator flagellins) has been reported, indicating both shared and disease-specific humoral immune patterns between CD and ME/CFS.^19^^,^^20^^,^^56^ Furthermore, prior PhIP-Seq studies in patients with ME/CFS demonstrated that antibodies against bacterial flagellins are significantly overrepresented, supporting a potential role of the humoral immune response against microbes in fatigue-related disease pathophysiology.^19^^,^^20^ In addition, AI-driven multi-omics modeling in ME/CFS has shown that fatigue is a heterogeneous symptom that must be interpreted carefully using clinical and biochemical parameters.^57^ Since anti-flagellin antibodies do not seem to be linked with IBD-associated fatigue in this study, that may suggest a distinct immune reactivity, and potentially different immune mechanisms underlying fatigue in IBD, compared to fatigue in ME/CFS. Confirmation in different cohorts with more precise fatigue assessment scores is needed to confirm these observations.

### Strengths of the study

This study leverages PhIP-Seq to comprehensively investigate systemic antibody responses underlying fatigue in patients with IBD following an unbiased approach. While previous studies utilizing PhIP-Seq were focused on the characterization of antibody repertoires of IMIDs such as IBD and ME/CFS in their entirety, this study shows that this technology can also be effectively used to address a more specific research question within clinical IBD focusing on complex multifactorial symptoms such as fatigue.^16^^,^^19^

Strengths of the study include the use of PhIP-Seq, which is a highly sensitive method for the identification of systemic antibody responses in fatigued patients with IBD. PhIP-Seq is cost-effective and high-throughput, enabling the analysis of hundreds of thousands of peptides at the same time. A broad rationally selected antigen repertoire was used, spanning microbes, food antigens, and immune (self-)antigens. It is essential to maintain a diverse antigen library to facilitate the discovery of potential findings, particularly in the identification of biomarkers. Another key strength of this study was the reporting of fatigue scores *within 24 h* of blood collection, which minimizes temporal confounders such as exposure to recent infections, thereby enhancing the reliability of the data in PhIP-Seq analysis. Another strength of this study is conducting sensitivity analyses twice, once in patients in remission to account for disease activity and anemia as confounders and a second time by comparing it to findings in patients with CFS/ME.

### Future perspective

IBD is a common multifactorial disease often presenting with nonspecific, hard-to-manage symptoms such as fatigue. Fatigue itself is also complex, multifactorial, and therefore difficult to comprehend. Little is known about the disease-specific mechanisms behind fatigue in IBD. Thus far, only subjective quantitative measures such as patient-reported scores exist. Treatment strategies revolve around managing disease activity, psychotherapy, and lifestyle measures like diet adjustments. Despite these efforts, the burden of fatigue for patients is still perceived as high. Given its complex nature, few studies attempt to investigate the pathophysiology of fatigue, specifically in IBD. Identifying changes in antibody prevalence offers an opportunity to better understand mechanisms of this symptom. Thereby, the most important contributing factors of fatigue in IBD can be identified and used to develop strategies to properly address it and eventually contribute to its management and prevention.

Future studies should incorporate longitudinal designs and more comprehensive characterization of relevant clinical factors to better disentangle their influence on antibody repertoires.

### Conclusion

In conclusion, this study suggests potential associations of specific systemic antibody responses with fatigue in patients with IBD. These findings support hypotheses on putative fatigue mechanisms that involve reactivities seemingly different from those in a disease like ME/CFS. Alterations were also dependent on disease activity, with differences between UC and CD, and can help understand symptom-specific pathophysiology. Although the analysis remained non-significant and it cannot be determined whether these findings are symptom- or disease-specific, these results still provide valuable insights, and this study shows the far-reaching potential of large data-driven analysis such as PhIP-Seq for improving symptom understanding and management.

### Limitations of the study

Several limitations of this study warrant consideration. Most importantly, this study lacked a well-defined control cohort with both fatigue score recordings and PhIP-Seq data, which precluded disentangling whether the observed antibody patterns relate to fatigue, underlying IBD, or general immune exposure history compared to those in healthy or non-IBD fatigue populations. Importantly, without appropriate control groups, these findings should not be interpreted as fatigue-specific immune signatures, but rather as associations observed within an IBD cohort stratified by fatigue levels. Furthermore, the definition of fatigue based on a one-dimensional Likert scale is predisposed to subjectivity, introduces measurement noise, and does not represent a validated multidimensional assessment of fatigue. This limited phenotypic resolution may have reduced the ability to detect robust associations and complicates the interpretation of underlying biological mechanisms. For example, the multidimensional fatigue inventory (MFI), which is a 20-item questionnaire that measures fatigue in 5 dimensions, including general, physical, motivation, and mental aspects, could be used in that regard.^58^ Other important influencing factors could be psychological comorbidities such as depression or sleep disturbances. In addition, residual confounding remains an important limitation. Although analyses were adjusted for age, sex, and smoking status, other relevant factors that may influence antibody repertoires were not available. These include specific medication use over time (e.g., other biologics, corticosteroids, immunomodulators), vaccination history, prior or recent infections, and disease duration. Such factors are particularly relevant for interpreting responses to common viral antigens such as EBV as well as influenza and may contribute to the observed patterns independently of fatigue. In particular, Anti-TNF therapies may interfere with B-cell activation and differentiation, effects on germinal center reactions, and alterations in antibody production, class switching, and long-term immune memory. Therefore, future studies should more comprehensively account for these factors, ideally through detailed longitudinal characterization of treatment exposure and integration into analytical models. While being high-throughput, PhIP-Seq cannot yet detect peptides longer than 100 amino acids, nor can it capture conformational structures, nor non-protein antigens like lipids and glycans. Given the large multiplicity (>300,000 peptides) relative to sample size (*n* = 327), peptide-level associations were underpowered, and no individual peptide remained significant after FDR correction. By aggregating peptides to biologically annotated taxonomies, several group-level differences were identified. These findings should be interpreted with caution, as aggregation-based approaches may capture patterns arising from correlated structure or random variation, and therefore require independent validation. To prevent overinterpretation, permutation analysis was conducted, which showed that these results could indeed be found at random. However, since these findings recurred across multiple analyses, and the study is designed as exploratory, these observations may help guide future investigation of immune processes relevant to fatigue in IBD but require confirmation in independent cohorts and orthogonal assays.

## Resource availability

### Lead contact

Further information and requests for resources and data should be directed to and will be fulfilled by the lead contact, Dr. Arno R. Bourgonje (a.r.bourgonje@umcg.nl).

### Materials availability

This study did not generate new unique reagents. Antibody-bound peptides used in this study are publicly available in the European Genome-Phenome Archive (EGA), and accession numbers are listed in the key resources table.

### Data and code availability

All data are incorporated into the article and its online supplemental information. PhIP-Seq data have been deposited at the European Genome-Phenome Archive as EGAS00001006999 and are available upon reasonable request to the University Medical Center Groningen (UMCG). Clinical data of the Groningen 1000IBD cohort have been deposited at the European Genome-Phenome Archive as EGAD00001003991. They are available upon request if access is granted. To request access, please contact the Department of Gastroenterology of the University Medical Center Groningen. All original code used for analyses in this study has been deposited at zenodo.org (for detailed links, see the key resources table in the STAR Methods section) and is publicly available as of the date of publication. Any additional information required to reanalyze the data reported in this paper is available from the lead contact upon request.

## Acknowledgments

The authors would like to thank all participants of the 1000IBD cohort. A.R.B. is supported by the EU Horizon Health consortium grant ID-DarkMatter-NCD (101136582) and the Innovation Health Initiative Joint Undertaking (IHI JU) grant INTERCEPT (101194780). T.V. is supported by the 10.13039/501100000781European Research Council (ERC Starting Grant, EarlyMicroAbs, project no. [P.N.] 101075733) and coordinates the Horizon Health consortium “ID-DarkMatter-NCD” (P.N.: 101136582). RKW is supported by the 10.13039/100015545Seerave Foundation, 10.13039/501100003246NWO, the Innovation Health Initiative Joint Undertaking (IHI JU) grant INTERCEPT (101194780) and the EU Horizon Health consortium grant ID-DarkMatter-NCD (101136582). Views and opinions expressed are, however, those of the author(s) only and do not necessarily reflect those of the European Union or the 10.13039/501100000781European Research Council. Neither the European Union nor the granting authority can be held responsible for them. T.V. gratefully acknowledges support from the project “Antibody repertoires against microbiota as biomarkers for ME/CFS” (Project no. 10091012110015), which is developed in the context of the ME/CFS Lines Consortium (P.N. 10091012110021). Both the project and the consortium are (partly) financed by the Netherlands Organization for Health Research and Development (ZonMw) as part of the ZonMw ME/CFS Research Programme. The funders had no role in study design, data collection and analysis, preparation of the manuscript or decision to publish.

## Author contributions

Conceptualization, A.R.B. and R.K.W.; investigation, M.G.G., T.V., R.K.W. and A.R.B.; methodology, M.G.G., T.V., A.R.B., and R.K.W.; funding acquisition, R.K.W.; supervision, R.K.W. and A.R.B.; writing – original draft, M.G.G.; writing - review and editing, all authors.

## Declaration of interests

A.R.B. has received a research grant from Janssen Pharmaceuticals and received speaker’s and advisory board fees from AbbVie and Ferring, outside the submitted work. GD received an unrestricted research grant from Takeda and speaker fees from Pfizer and Janssen Pharmaceuticals. RKW acted as consultant for Takeda, received unrestricted research grants from Takeda, Johnson & Johnson, Tramedico, and Ferring, and received speaker’s fees from MSD, AbbVie, and Janssen. M.C.V. has received speaker’s fees from AbbVie, Janssen-Cilag, Alpha Sigma, and Ferring B.V. Pharmaceuticals.

All other authors have no conflicts of interest to declare.

## Declaration of generative AI and AI-assisted technologies in the writing process

During the preparation of this work, the authors used ChatGPT (OpenAI) to assist with improving the structure and readability of the manuscript and to support the refinement of Python scripts used for statistical analyses. All content generated with the assistance of these tools was critically reviewed, validated, and edited by the authors. The authors take full responsibility for the content of this publication.

## STAR★Methods

### Key resources table

**Table:** 

REAGENT or RESOURCE	SOURCE	IDENTIFIER
**Bacterial and virus strains**		

T7Select 10-3 cloning kit	Merck	Cat#70550-3

**Biological samples**		

327 serum samples and clinical metadata from individuals with IBD included in the present study	Imhann et al.^59^, Bourgonje et al.^16^	https://doi.org/10.1186/s12876-018-0917-5 https://doi.org/10.1016/j.immuni.2023.04.003

**Chemicals, peptides, and recombinant proteins**		

IPEGAL CA 630	Sigma-Aldrich	Cat#I3021
Protein A magnetic beads	Thermo Fisher Scientific	Cat#10008D
Protein G magnetic beads	Thermo Fisher Scientific	Cat#10009D
Q5 polymerase	New England Biolabs	Cat#M0493L
Bovine Serum Albumin, heat shock fraction, pH 7, R98%	Sigma-Aldrich / Merck	Cat#A7906-100G

**Critical commercial assays**		

QIAquick gel extraction kit	Qiagen	Cat#28704
QIAquick PCR purification kit	Qiagen	Cat#28104

**Deposited data**		

Raw data for the PhIP-Seq experiments	Bourgonje et al.^16^	EGA: EGAS00001006999 https://doi.org/10.1016/j.immuni.2023.04.003

**Oligonucleotides**		

PCR1	tcgtcggcagcgtcagatgtgtataagagacag GTTACTCGAGTGCGGCCGCAAGC	N/A
	gtctcgtgggctcggagatgtgtataagagacag ATGCTCGGGGATCCGAATTC	N/A
PCR2	Illumina Nextera combinatorial dual index primers	N/A
PCR3	AATGATACGGCGACCACCGA	N/A
	CAAGCAGAAGACGGCATACGA	N/A
R1 primer	ttactcgagtgcggccgcaagctttca	N/A
R2 primer	tgtgtataagagacagatgctcggggatccgaattct	N/A
Library amplification primer fwd	GATGCGCCGTGGGAATTCT	N/A
Library amplification primer rev	GTCGGGTGGCAAGCTTTCA	N/A

**Recombinant DNA**		

Oligo pool (200 mers)	Twist Bioscience	N/A
Oligo pool (230 mers)	Agilent Technologies	N/A

**Software and algorithms**		

Peptide quantification and enrichment determination	Vogl et al.^19^ and Leviatan et al.^25^	https://zenodo.org/records/7307894 https://doi.org/10.1126/sciadv.abq2422 https://doi.org/10.1016/j.immuni.2022.11.004
Python programming language	Python Software Foundation	v.3.10.9; https://www.python.org
pandas	The pandas development team	v.1.5.3; https://pandas.pydata.org
NumPy	NumPy Developers	v.1.23.5; https://numpy.org
statsmodels	statsmodels Developers	v.0.13.5; https://www.statsmodels.org
seaborn	Waskom	v.0.12.2; https://seaborn.pydata.org
matplotlib	Matplotlib Developers	v.3.7.0; https://matplotlib.org
Descriptive statistics, association analyses, logistic regressions	This paper	https://doi.org/10.5281/zenodo.18651941

**Other**		

Freedom Evo liquid handling robot	Tecan	N/A
MASTERBLOCK, 96w, PP, 2ml, Natural, 50/case	Danyel biotech	Cat#60-780270
Corning Axygen AM-2ML-SQ AxyMat	Biolab Ltd	Cat#AXY-AM-2ML-SQ

### Experimental model and study participant details

This study was a retrospective analysis of prospectively collected observational cohort data from human participants with established inflammatory bowel disease (IBD) who were enrolled in the 1000IBD cohort at the University Medical Center Groningen (UMCG), the Netherlands, between 2010 and 2019. The study included human participants only. No animal models, plants, microbe strains, cell lines, or primary cell cultures were used. Accordingly, animal maintenance/care, cell line authentication, and mycoplasma testing were not applicable.

Inclusion in the present study was based on the availability of both fatigue scores and phage-display immunoprecipitation sequencing (PhIP-Seq) data, resulting in a study population of 327 patients. No additional inclusion or exclusion criteria were applied. No formal sample size calculation was performed because the sample size was determined by the availability of both fatigue-score data and PhIP-Seq data. Participants were adult patients with established IBD. Age at serum sampling was recorded and is reported in Tables S1, S2, S3, and S4. The cohort consisted of patients with Crohn’s disease (CD; *n* = 156, 47.7%) and ulcerative colitis (UC; *n* = 171, 52.3%). The study population included both male and female participants, with a higher proportion of women (*n* = 198, 60.6%). Genotype was not assessed as part of this study. Data on gender, ancestry, race, ethnicity, and socioeconomic status were not available.

Participants were not assigned to experimental treatment groups. For the present analyses, participants were allocated to predefined fatigue groups based on self-reported fatigue scores, including quartile-based comparisons. The highest quartile (Q4) represented the most fatigued patients, while the remaining quartiles (<Q4) served as the primary comparator group. Additional predefined contrasts included Q1 versus Q4, Q1+Q2 versus Q3+Q4, and Q1 versus >Q1. Laboratory processing of PhIP-Seq samples was performed without using fatigue status for sample allocation or interpretation.

Sex was recorded at the time of serum sampling and was included as a covariate in the multivariable analyses together with age and smoking status. As reported in the Results and Tables S1, S2, S3, and S4, sex was associated with fatigue scores in this cohort. Because gender, ancestry, race, ethnicity, and socioeconomic status were not available, we could not assess whether these factors influenced fatigue-associated antibody profiles. This limits the generalizability of our findings, as these factors may be associated with fatigue perception, symptom reporting, comorbidities, environmental exposures, healthcare access, and immune variation. Future studies with more detailed demographic, clinical, and socioeconomic data are needed to determine whether the observed antibody patterns are consistent across diverse patient groups and whether they are specific to fatigue in inflammatory bowel disease.

Fatigue scores were assessed using a patient-reported 10-point Likert scale completed within 24 hours of blood collection. Data were anonymized before analysis and analyzed without directly identifiable patient information. The study was approved by the institutional medical ethical review board of the University Medical Center Groningen (approval number 08/338), and all participants provided written informed consent. This study was not a clinical trial; therefore, no clinical trial registry number is applicable.

#### Ethical approval

The study was approved by the institutional medical ethical review board of the University Medical Center Groningen (approval number 08/338), and all participants provided written informed consent.

### Method details

#### Fatigue assessment and clinical definitions

Fatigue was assessed using a patient-reported 10-point Likert scale completed within 24 hours of blood collection.^8^ The question was: “How fatigued has the patient been during the last 24 hours?”, in Dutch: “In hoeverre heeft u last gehad van vermoeidheid in de afgelopen 24 uur?” Fatigue scores were analyzed both as continuous values and as categorical variables using predefined quartile-based groupings. The highest quartile (Q4) represented the most fatigued patients, while the remaining quartiles (< Q4) served as the primary comparator group. Additional fatigue contrasts were explored, including Q1 versus Q4, Q1+Q2 versus Q3+Q4, and Q1 versus > Q1, as specified in the results section.

Clinical disease activity was assessed using the Harvey-Bradshaw Index (HBI) for Crohn’s disease and the Simple Clinical Colitis Activity Index (SCCAI) for ulcerative colitis. Clinical remission was defined as HBI ≤ 5 for Crohn’s disease or SCCAI < 2.5 for ulcerative colitis.^60^ Biochemical remission was defined as a C-reactive protein (CRP) level <5 mg/L. Anemia was defined according to Dutch reference ranges as hemoglobin < 8.5 mmol/L for males and < 7.5 mmol/L for females.^61^ For sensitivity analyses, a sub-cohort of patients without anemia and in clinical and biochemical remission was analyzed separately to reduce confounding by disease activity and systemic inflammation.

#### PhIP-Seq library design and workflow

Systemic antibody responses were profiled using PhIP-Seq, a high-throughput sequencing-based method that enables parallel detection of serum antibodies against large libraries of peptide antigens.^16^^,^^24^^,^^25^ This study used previously generated PhIP-Seq data from the 1000IBD cohort.^16^ The antigen library used in this study consisted of approximately 344,000 rationally selected peptide antigens, derived from two complementary libraries. One library consisted of approximately 244,000 microbiota enriched peptide antigens, while the second library consisted of approximately 100,000 peptide antigens covering phages, immune epitopes, and allergens.^16^^,^^24^^,^^25^

The combined antigen library covered a broad range of antigenic sources, including viruses, pathogenic and commensal bacteria, autoantigens, allergens, virulence factors, and control peptides.^16^^,^^23^^,^^24^^,^^25^ Antigens were rationally selected to represent microbial, viral, food-related, immune-related, and control antigens, with enrichment for proteins likely to be exposed to the host immune system, such as membrane proteins, secreted proteins, motility proteins, and virulence factors.^24^^,^^25^ Peptide-coding oligonucleotides were cloned into T7 phage libraries, enabling identification of antibody-bound peptide antigens by sequencing.^24^^,^^25^ A schematic overview of the library content is provided in Figure 2B.

In brief, peptide-coding oligonucleotide pools from Twist Bioscience and Agilent Technologies were cloned into T7 phage libraries using the T7Select 10-3 cloning system. Circulating antibodies from patient serum were incubated with the phage-displayed peptide library in buffer containing bovine serum albumin and IPEGAL CA 630, followed by immunoprecipitation of antibody-phage complexes using protein A and protein G magnetic beads. Bead washing was performed using a Freedom Evo liquid-handling robot with 96-well MASTERBLOCK plates and AxyMat sealing mats. Bound phages were amplified by polymerase chain reaction using Q5 polymerase, library amplification primers, and Illumina amplicon sequencing primers. Polymerase chain reaction products were purified using QIAquick gel extraction and polymerase chain reaction purification kits, and peptide identities were determined using paired-end next-generation sequencing. Sequencing reads were mapped to peptide identifiers to reconstruct individual antibody epitope repertoires. The PhIP-Seq workflow is summarized in Figure 2A.

#### PhIP-Seq data preprocessing

PhIP-Seq data preprocessing and normalization followed the previously established pipeline used for the 1000IBD PhIP-Seq dataset.^16^ Sequencing reads were downscaled to a uniform depth of 1.25 million identifiable reads per sample where possible. A minimum threshold of 750,000 identifiable reads was required for downstream analysis. Peptide enrichment was quantified by comparing post-immunoprecipitation read counts with pre-immunoprecipitation input-library read counts. For each peptide, enrichment was modeled relative to the input library using a generalized Poisson model.^16^^,^^24^ Peptide-level *p* values were adjusted for multiple testing using Bonferroni correction, and peptides with adjusted *p* ≤ 0.05 were considered seropositive.

For the present fatigue analyses, the PhIP-Seq-derived antibody data were analyzed as a binary peptide-reactivity matrix. Group-specific antibody prevalences were calculated as the proportion of patients within each fatigue group who were seropositive for a given peptide. Prevalence ratios and prevalence-based fold changes were then calculated by comparing antibody prevalence between fatigue groups for each predefined contrast. To reduce the influence of rare, highly individual-specific, or near-ubiquitous antibody signals, peptides were filtered based on prevalence across the cohort. Only peptides present in at least 5% and at most 95% of participants were retained for statistical analysis, resulting in 2,550 antibody bound peptides for downstream analyses.

#### Peptide aggregation and grouping strategy

To address the high dimensionality of the PhIP-Seq dataset relative to the sample size, a biologically informed aggregation strategy was applied to identify coordinated antibody response patterns. The analysis was initiated at the individual peptide level using multivariable logistic regression across all retained peptides. Although no single peptide remained statistically significant after multiple testing correction, exploratory inspection of the highest-ranking peptides suggested recurring patterns across specific biological categories, including Epstein-Barr virus (EBV), influenza viruses, herpesviruses more broadly, and *Staphylococcus aureus*.

Based on these recurring patterns, peptides were grouped using library annotations and external identifiers, including UniProt, the Immune Epitope Database (IEDB), and the Virulence Factor Database (VFDB).^16^^,^^24^^,^^25^ Grouping was performed in a hierarchical manner to evaluate the level at which signals were most consistent. First, each library and large taxonomic groups (e.g., IEDB, VFDB, bacteria, viruses, etc.) were grouped to see if there are library-specific signals. Then antibodies were aggregated into less broad taxonomic or biological categories, such as herpesviruses, influenza viruses, and bacterial taxa. Subsequently, more specific groupings were examined within these categories, such as EBV within herpesviruses. For selected antigens showing consistent patterns, subunit-level analyses were performed, including EBV latent proteins such as EBNA-1 and EBNA-3 and lytic proteins such as EA-D/BMRF1.

When peptides mapped to multiple proteins, the canonical library mapping was used. Peptides that could not be confidently assigned to a specific subunit were excluded from subunit-level summaries. To account for redundancy due to overlapping peptides targeting the same antigen, aggregation at the group level was performed by summarizing peptide-level prevalence ratios or fold changes using the median. This approach provided a robust central estimate of antibody reactivity while reducing the influence of outlier peptides. These group aggregation analyses were exploratory and were interpreted as hypothesis-generating.

#### Statistical analysis

Baseline characteristics of the study population were presented as means with standard deviations (SDs), medians with interquartile ranges (IQRs), or counts with percentages (%), as appropriate. Data normality was assessed using normal probability Q-Q plots and histograms. Normality was formally tested using the Shapiro-Wilk test. Baseline characteristics were compared between fatigue groups using independent t-tests, Mann-Whitney U tests, chi-squared tests, or Fisher’s exact tests, as appropriate. Spearman’s rank correlation coefficients were calculated to assess associations between fatigue scores and disease activity parameters or hemoglobin values. These checks were used to determine whether parametric or non-parametric tests were appropriate.

Associations between fatigue status and antibody responses were assessed using multivariable logistic regression models adjusted for age, sex, and smoking status. Antibody data were analyzed as a binary peptide-reactivity matrix. Analyses were performed across predefined fatigue contrasts: Q4 versus < Q4 as the primary contrast, Q4 versus Q1, Q1+Q2 versus Q3+Q4, and Q1 versus > Q1. Each contrast was repeated in the full cohort, by IBD diagnosis (Crohn’s disease and ulcerative colitis), and in the remission sub-cohort. The remission sub-cohort included patients in clinical remission, with CRP < 5 mg/L, and without anemia as defined above.

Prior to regression modeling, age and smoking were standardized, and sex was coded as a binary variable. Multicollinearity among covariates was assessed using variance inflation factors, applying a threshold of 10. Peptide-level associations were evaluated using logistic regression models adjusted for age, sex, and smoking status. For each peptide, regression coefficients, *p values*, and confidence intervals corresponding to fatigue-group status were extracted. In addition, group-specific antibody prevalences and prevalence-based fold changes were calculated for descriptive comparison and downstream aggregation analyses.

Multiple testing was controlled using the Benjamini-Hochberg false discovery rate procedure. Associations with a false discovery rate-adjusted *q* value < 0.1 were considered statistically significant, while *p* values ≤ 0.05 were considered nominally significant. For group analyses, peptide-level antibody prevalence ratios were compared between fatigue strata using two-sided Wilcoxon rank-sum tests, and multiple testing was controlled within each contrast family using the Benjamini-Hochberg procedure.

Permutation-based analyses were performed to assess whether observed aggregation-level patterns exceeded random structure. Fatigue group labels were randomly reassigned across patients while preserving group sizes, and predefined aggregated statistics based on peptide-level prevalence ratios were recalculated for each permutation. In the full cohort, a global statistic was computed as the maximum absolute aggregated group-level effect across predefined antigen groups. In addition, targeted permutation analyses were performed for selected signals highlighted in the manuscript. Empirical *p* values were obtained by comparing the observed aggregated statistic with its permutation-derived null distribution.

A sensitivity analysis was performed using the antigen set from previous studies on systemic antibody responses against microbiota flagellins in chronic fatigue syndrome and Crohn’s disease.^19^^,^^20^ This analysis was performed to evaluate whether previously reported flagellin-associated antibody patterns were also associated with fatigue in the present IBD cohort.

Statistical parameters, including the exact value of *n*, what *n* represents, *p* values, false discovery rate-adjusted *q* values, regression coefficients, confidence intervals, and descriptive antibody prevalences, are reported in the Results, figure legends, and Tables S1, S2, S3, S4, S5, S6, S7, S8, S9, S10, S11, S12, S13, S14, S15, S16, S17, S18, S19, S20, S21, S22, S23, S24, S25, S26, S27, S28, S29, S30, S31, S32, S33, S34, S35, S36, S37, S38, S39, and S40. In the supplementary tables, *n* represents the number of patients included in the respective fatigue contrast, disease subgroup, or remission subgroup. Measures of variability are reported as SDs or IQRs, as indicated in the relevant table legends.

Items listed in the key resources table are mentioned in the method details or main text where relevant. These include resources used for PhIP-Seq library generation, immunoprecipitation, amplification, sequencing, peptide quantification and enrichment determination, deposited PhIP-Seq data, and the statistical analysis code used in the present study.

Data analysis was performed using the Python programming language (v.3.10.9, Python Software Foundation, https://www.python.org), using pandas (v.1.5.3), NumPy (v.1.23.5), and statsmodels (v.0.13.5). Data visualization was performed using seaborn (v.0.12.2) and matplotlib (v.3.7.0).

## Supplemental information

Table S1. Demographic and clinical characteristics of the study population comparing high fatigue (Q4) with low-to-moderate fatigue (<Q4)

Table S2. Demographic and clinical characteristics of the study population comparing high fatigue (Q4) with low fatigue (Q1)

Table S3. Demographic and clinical characteristics of the study population comparing higher fatigue (Q3+Q4) with lower fatigue (Q1+Q2)

Table S4. Demographic and clinical characteristics of the study population comparing patients above the lowest fatigue quartile (>Q1) with patients in the lowest fatigue quartile (Q1)

Table S5. Logistic regression results for the association between individual antibody reactivity and fatigue status in the full IBD cohort comparing high fatigue (Q4) with low-to-moderate fatigue (<Q4)

Table S6. Logistic regression results for the association between individual antibody reactivity and fatigue status in the UC cohort comparing high fatigue (Q4) with low-to-moderate fatigue (<Q4)

Table S7. Logistic regression results for the association between individual antibody reactivity and fatigue status in the CD cohort comparing high fatigue (Q4) with low-to-moderate fatigue (<Q4)

Table S8. Logistic regression results for the association between individual antibody reactivity and fatigue status in the remission cohort comparing high fatigue (Q4) with low-to-moderate fatigue (<Q4)

Table S9. Logistic regression results for the association between individual antibody reactivity and fatigue status in the remission UC cohort comparing high fatigue (Q4) with low-to-moderate fatigue (<Q4)

Table S10. Logistic regression results for the association between individual antibody reactivity and fatigue status in the remission CD cohort comparing high fatigue (Q4) with low-to-moderate fatigue (<Q4)

Table S11. Logistic regression results for the association between individual antibody reactivity and fatigue status in the full IBD cohort comparing high fatigue (Q4) with low fatigue (Q1)

Table S12. Logistic regression results for the association between individual antibody reactivity and fatigue status in the UC cohort comparing high fatigue (Q4) with low fatigue (Q1)

Table S13. Logistic regression results for the association between individual antibody reactivity and fatigue status in the CD cohort comparing high fatigue (Q4) with low fatigue (Q1)

Table S14. Logistic regression results for the association between individual antibody reactivity and fatigue status in the remission cohort comparing high fatigue (Q4) with low fatigue (Q1)

Table S15. Logistic regression results for the association between individual antibody reactivity and fatigue status in the remission UC cohort comparing high fatigue (Q4) with low fatigue (Q1)

Table S16. Logistic regression results for the association between individual antibody reactivity and fatigue status in the remission CD cohort comparing high fatigue (Q4) with low fatigue (Q1)

Table S17. Logistic regression results for the association between individual antibody reactivity and fatigue status in the full IBD cohort comparing higher fatigue (Q3+Q4) with lower fatigue (Q1+Q2)

Table S18. Logistic regression results for the association between individual antibody reactivity and fatigue status in the UC cohort comparing higher fatigue (Q3+Q4) with lower fatigue (Q1+Q2)

Table S19. Logistic regression results for the association between individual antibody reactivity and fatigue status in the CD cohort comparing higher fatigue (Q3+Q4) with lower fatigue (Q1+Q2)

Table S20. Logistic regression results for the association between individual antibody reactivity and fatigue status in the remission cohort comparing higher fatigue (Q3+Q4) with lower fatigue (Q1+Q2)

Table S21. Logistic regression results for the association between individual antibody reactivity and fatigue status in the remission UC cohort comparing higher fatigue (Q3+Q4) with lower fatigue (Q1+Q2)

Table S22. Logistic regression results for the association between individual antibody reactivity and fatigue status in the remission CD cohort comparing higher fatigue (Q3+Q4) with lower fatigue (Q1+Q2)

Table S23. Logistic regression results for the association between individual antibody reactivity and fatigue status in the full IBD cohort comparing patients above the lowest fatigue quartile (>Q1) with patients in the lowest fatigue quartile (Q1)

Table S24. Logistic regression results for the association between individual antibody reactivity and fatigue status in the UC cohort comparing patients above the lowest fatigue quartile (>Q1) with patients in the lowest fatigue quartile (Q1)

Table S25. Logistic regression results for the association between individual antibody reactivity and fatigue status in the CD cohort comparing patients above the lowest fatigue quartile (>Q1) with patients in the lowest fatigue quartile (Q1)

Table S26. Logistic regression results for the association between individual antibody reactivity and fatigue status in the remission cohort comparing patients above the lowest fatigue quartile (>Q1) with patients in the lowest fatigue quartile (Q1)

Table S27. Logistic regression results for the association between individual antibody reactivity and fatigue status in the remission UC cohort comparing patients above the lowest fatigue quartile (>Q1) with patients in the lowest fatigue quartile (Q1)

Table S28. Logistic regression results for the association between individual antibody reactivity and fatigue status in the remission CD cohort comparing patients above the lowest fatigue quartile (>Q1) with patients in the lowest fatigue quartile (Q1)

Table S29. Logistic regression results for the association between fatigue status and antibodies included in Vogl et al. ^19^ in the full IBD cohort comparing high fatigue (Q4) with low-to-moderate fatigue (<Q4)

Table S30. Logistic regression results for the association between fatigue status and antibodies included in Vogl et al. ^19^ in the UC cohort comparing high fatigue (Q4) with low-to-moderate fatigue (<Q4)

Table S31. Logistic regression results for the association between fatigue status and antibodies included in Vogl et al. ^19^ in the CD cohort comparing high fatigue (Q4) with low-to-moderate fatigue (<Q4)

Table S32. Logistic regression results for the association between fatigue status and antibodies included in Vogl et al.19 in the remission cohort comparing high fatigue (Q4) with low-to-moderate fatigue (<Q4)

Table S33. Logistic regression results for the association between fatigue status and antibodies included in Vogl et al. ^19^ in the remission UC cohort comparing high fatigue (Q4) with low-to-moderate fatigue (<Q4)

Table S34. Logistic regression results for the association between fatigue status and antibodies included in Vogl et al. ^19^ in the remission CD cohort comparing high fatigue (Q4) with low-to-moderate fatigue (<Q4)

Table S35. Logistic regression results for the association between fatigue status and flagellin-targeting antibodies from Vogl et al. ^19^ in the full IBD cohort comparing high fatigue (Q4) with low-to-moderate fatigue (<Q4)

Table S36. Logistic regression results for the association between fatigue status and flagellin-targeting antibodies from Vogl et al. ^19^ in the UC cohort comparing high fatigue (Q4) with low-to-moderate fatigue (<Q4)

Table S37. Logistic regression results for the association between fatigue status and flagellin-targeting antibodies from Vogl et al. ^19^ in the CD cohort comparing high fatigue (Q4) with low-to-moderate fatigue (<Q4)

Table S38. Logistic regression results for the association between fatigue status and flagellin-targeting antibodies from Vogl et al. ^19^ in the remission cohort comparing high fatigue (Q4) with low-to-moderate fatigue (<Q4)

Table S39. Logistic regression results for the association between fatigue status and flagellin-targeting antibodies from Vogl et al. ^19^ in the remission UC cohort comparing high fatigue (Q4) with low-to-moderate fatigue (<Q4)

Table S40. Logistic regression results for the association between fatigue status and flagellin-targeting antibodies from Vogl et al. ^19^ in the remission CD cohort comparing high fatigue (Q4) with low-to-moderate fatigue (<Q4)
